# Dietary supplementation with natto fermented by *Bacillus subtilis* var. *natto* strain TTCC903 alleviates heat stress and improves egg production in aged laying hens

**DOI:** 10.1016/j.psj.2026.107039

**Published:** 2026-05-01

**Authors:** Kaori Suzuki, Hikaru Ikarugi, Takanobu Nishikawa, Ryosuke Kadoya

**Affiliations:** a Department of Natto Research and Development, Takanofoods Co., Ltd, 1542, Noda, Omitama City, Ibaraki 311-3411, Japan; b Department of Livestock Research and Development, Takanofoods Co.,Ltd, 1542, Noda, Omitama City, Ibaraki 311-3411, Japan; c Department of Food and Nutrition, School of Life Studies, Sugiyama Jogakuen University, 17-3 Hoshigaoka motomachi, Chikusa-ku, Nagoya, Aichi, 464-8662, Japan

**Keywords:** Aged laying hens, Heat stress, Probiotics, Egg production, *Bacillus subtilis*

## Abstract

Heat stress is a major constraint on poultry production, particularly in aged laying hens with diminished thermoregulatory and metabolic capacity. Probiotics, including *Bacillus* species, have been proposed as dietary supplements to mitigate these effects. This study investigated whether supplementation with natto fermented by *Bacillus subtilis* var. *natto* strain TTCC903 alleviates heat stress and improves productivity in aged laying hens. Ambient conditions indicated mild to moderate heat stress throughout the trial. In controls, blood malondialdehyde (MDA) increased by 26 %, whereas natto-supplemented groups maintained stable or reduced levels, with the 0.05 % supplementation group showing a 29 % reduction. The hen-housed egg production rate (HHEP) in controls averaged 87.4 %, while the 0.05 % and 0.5 % supplementation groups averaged 89.5 % and 90.8 %, respectively (*p* < 0.05), with the 0.5 % group exceeding 92.8 % during weeks of high THI. Egg production expressed as g/hen/day tended to increase in natto-fed hens, and the feed conversion ratio improved significantly in the 0.5 % group (1.60 vs. 1.69 in controls). The defective egg rate was ≤0.5 % across all groups but showed a relative 50 % reduction in the 0.5 % group. Eggshell strength was preserved in natto-fed hens but declined in controls. Natto supplementation also reduced diarrhea incidence (4 % vs 9 %) and restored the ileal villus-to-crypt ratio (5.00 vs. 3.76 in controls). In conclusion, supplementation with natto fermented by *B. subtilis* var. *natto* strain TTCC903 mitigated oxidative and physiological responses to heat stress, improved egg production efficiency, and enhanced intestinal health in aged hens. These findings extend previously reported benefits of this strain in fish and ruminants to poultry, supporting its potential as a probiotic technology in the livestock industry.

## Introduction

Global warming has emerged as a critical worldwide issue, profoundly affecting primary industries such as agriculture and livestock production (Cheng et al., 2022). Rising average temperatures, particularly the increasing frequency of heatwaves, expose livestock to more severe high-temperature environments, thereby elevating the risk of heat stress. Because livestock have limited thermoregulatory capacity, ambient temperature increases readily impair physiological functions and productivity (Das*,* et al., 2016). Consequently, heat stress reduces feed intake, suppresses growth and egg production, lowers reproductive efficiency, and compromises immune function. The accompanying rise in oxidative stress further heightens disease susceptibility and, in severe cases, can lead to mortality (Aryal*,* et al., 2025; Wasti*,* et al., 2020). From an industrial perspective, these physiological impairments reduce production efficiency and product quality while increasing veterinary and management costs (Oke*,* et al., 2024). The impact of heat stress is especially pronounced in high-yielding livestock, such as dairy cows and laying hens, and poses a serious threat to the sustainability of the livestock industry.

Among these, heat stress in older laying hens presents a particularly serious challenge. Age-related declines in metabolic function and thermoregulatory capacity reduce their tolerance to high temperatures, resulting in lower egg production and eggshell quality compared with younger birds (Gonzalez*,* et al., 2025). Nevertheless, the poultry industry requires the long-term use of older hens for economic reasons, making their effective management a critical issue. Recent studies have reported that both management strategies (e.g., housing modifications) and nutritional interventions can improve production performance, egg quality (e.g., Haugh unit values), and feed intake in aged laying hens (Onbasilar*,* et al., 2020). Current on-farm countermeasures include ventilation, shading, mist spraying, and cold water provision, as well as feed adjustments and the use of functional additives (Nawaz*,* et al., 2021; Saeed*,* et al., 2019). While these multifaceted measures are important for sustaining survival and productivity, the progression of climate change suggests that existing approaches alone are insufficient, highlighting the urgent need for new strategies to maintain the health and productivity of older hens under heat stress conditions (Cheng*,* et al., 2022).

One promising approach is dietary supplementation with probiotics, defined as “live microorganisms that confer a health benefit on the host (Binda*,* et al., 2020; Hill*,* et al., 2014).” Probiotics have been reported to improve intestinal microbiota balance, inhibit colonization of pathogenic bacteria, enhance nutrient utilization, and modulate immune responses (Halder*,* et al., 2024; Naeem and Bourassa, 2025). In poultry, the use of probiotics—primarily lactic acid bacteria and *Bacillus* species—has been shown to improve the feed conversion ratio (FCR), increase egg production and quality, and strengthen the intestinal barrier function (Cufadar*,* et al., 2024). Furthermore, recent studies suggest that probiotics may mitigate heat stress by enhancing antioxidant enzyme activity and suppressing inflammatory cytokines, thereby preventing declines in productivity under hot environmental conditions (Hashemitabar and Hosseinian, 2024).

Despite these advances, the effects of probiotics in older hens remain poorly understood. During the late laying period, age-related declines in metabolism and immunity heighten susceptibility to heat stress, making it difficult to sustain production efficiency (Attia*,* et al., 2020). Clarifying how probiotics alleviate heat stress and support productivity and health in older hens is therefore of critical importance from both academic and industrial perspectives. This study focused on *Bacillus subtilis* var. *natto* as a probiotic of interest (Ruiz Sella*,* et al., 2021). *B. subtilis* var. *natto*, the primary fermenting organism in natto, a traditional Japanese food, forms spores that confer high heat resistance and stability, making it suitable as a feed additive (Cutting, 2011). Previous studies have reported that extracts from *B. subtilis* var. *natto* strain TTCC903 inhibit SARS-CoV-2 infection in cultured cells and prevent bovine herpesvirus 1 infection, highlighting its antiviral and immunomodulatory properties (Oba*,* et al., 2021), as well as suppressing infection in mice caused by the Aujeszky’s disease virus (Kobayashi*,* et al., 2023). Dietary supplementation with natto fermented by *B. subtilis* var. *natto* strain TTCC903 increased growth rates and reduced cortisol levels in carp under high-temperature conditions (Niwa*,* et al., 2026). These findings suggest that natto-based probiotics may also benefit poultry. Accordingly, the present study aimed to evaluate the effects of dietary supplementation with natto bacteria in older laying hens and to examine their potential for mitigating heat stress and maintaining productivity.

## Materials and methods

### Laying hens and experimental design

A total of 135 laying hens of the White Leghorn breed (Julia, aged 498 days) were used.

Three treatment groups were established: a control group fed the control diet (Table 1), and two experimental groups fed the control diet supplemented with either 0.05 % or 0.5 % dried fermented soybean powder (natto) fermented with the TTCC903 strain, respectively. The feed composition was calculated based on Standard Tables of Feed Composition in Japan (2009).

**Table 1:** Feed ingredients and mixing ratio (%).

Feed Ingredient	Inclusion Levels (%)
Corn	52.32
Grain sorghum	5.00
Soybean meal	22.30
Fish meal (CP65)	2.80
Corn gluten meal (CP60)	2.60
Vegetable oil	2.50
Dicalcium phosphate	1.20
Calcium carbonate	10.50
Salt	0.33
DL-methionine	0.18
Vitamin-mineral premix	0.25
Selenium	0.02
Total	100

**Fig. 1:**
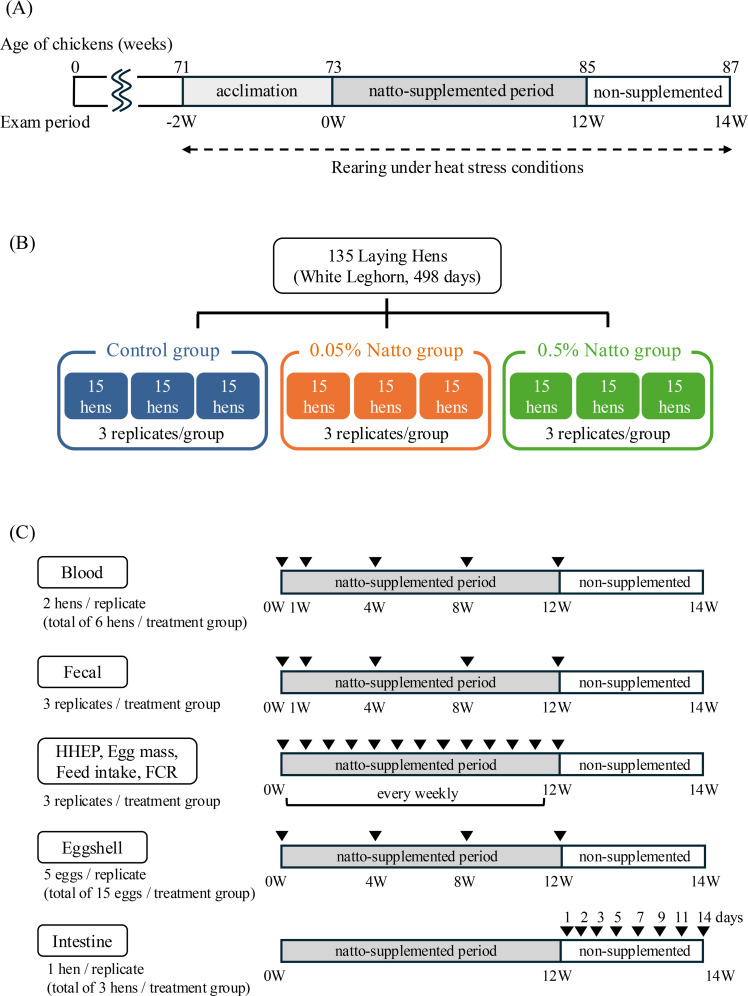
Experimental design and sampling scheme. (A) Timeline of the experiment. Laying hens were acclimated for 2 weeks prior to the start of the feeding trial (0 W), followed by a 12-week natto-supplemented period and a 2-week non-supplemented period under heat-stress conditions. (B) Allocation of experimental groups. A total of 135 aged laying hens (White Leghorn, 498 days old) were randomly assigned to three treatment groups (control, 0.05 % natto, and 0.5 % natto), each consisting of three replicates with 15 hens per replicate. (C) Sampling scheme for each analysis. Black triangles indicate sampling points. Blood samples were collected from two hens per replicate (total of six hens per treatment group) at 0, 1, 4, 8, and 12 weeks, and the same individuals were repeatedly sampled. Fecal samples were collected at multiple time points from each replicate (three replicates per treatment group). Egg production parameters (hen-housed egg production, egg mass, feed intake, and feed conversion ratio) were calculated using three replicates per treatment group. Eggshell strength was evaluated using five eggs per replicate (total of 15 eggs per treatment group) at the indicated time points. Intestinal morphology was evaluated in one hen per replicate (total of three hens per treatment group) at the indicated time points.

The hens were randomly allocated to the three treatment groups, with three replicates per treatment and 15 hens per replicate, to ensure a nearly uniform distribution of laying rates. After a 2-week pre-treatment period with continuous feeding of the control diet, the hens were fed the respective experimental diets for 12 weeks. Subsequently, they were switched back to the control diet for a final 2-week period (Fig. 1). The hens were individually housed in laying hen cages installed in a forced-ventilation, windowless poultry house. Water was provided ad libitum, and lighting was managed with a 14-hour light period (6:00 to 20:00) and a 10-hour dark period. To prevent contamination, a vinyl sheet was placed between the control and the natto-supplemented groups. To simulate a hot environment, floor heating and stoves were used to maintain a room temperature of approximately 30°C.

### Index of heat stress

During the test period, room temperature and humidity in the poultry house were measured twice daily at 8:00 and 16:00 using a temperature and humidity data logger (ST-50A, Sekonic Co., Ltd.). Environmental heat stress was evaluated based on temperature and the temperature–humidity index (THI). For laying hens, the thermoneutral range is generally reported to be approximately 18–24°C, with increasing heat load observed at 25–29°C and clear heat-stress responses occurring at around 30°C (Ahmad*,* et al., 2022; Kim*,* et al., 2022; Wang*,* et al., 2020). THI for laying hens has been reported to fall into four categories: comfort (THI < 70), alert (70–75), danger (76–81), and emergency (> 81) (Kim*,* et al., 2020). The THI was calculated for each time point from the obtained temperature and humidity data. The formula for calculating THI is shown below.

THI=(0.8×temperature+(relativehumidity/100)×(temperature−14.4))+46.4

### Blood sampling and lipid peroxidation measurement

Blood samples were collected at five time points: at the start of test diet feeding, and at the end of week 1, 4, 8, and 12. At the first time point, two hens per replicate (total of six hens per treatment group) were selected, and blood samples were subsequently collected from the same individuals at each sampling time. Individual hens were treated as experimental units for blood parameter analysis, and the same individuals were repeatedly sampled over time. Samples were stored at −80°C until analysis. Plasma malondialdehyde (MDA) concentrations were determined using a TBARS Assay Kit (Cayman Chemical), with measurements performed in duplicate. Absorbance was measured using a microplate reader (Infinite® M NANO, TECAN), and MDA concentrations were calculated from a standard calibration curve.

### Fecal sampling and natto bacteria count measurement

Fecal–urine mixtures (excluding cecal feces) were collected from each replicate at the same five time points as blood sampling, as well as at eight additional time points: 1, 2, 3, 5, 7, 9, 11, and 14 days after the end of test feeding. Each sample was diluted 10-fold with physiological saline to prepare a stock suspension. The suspension was then heat-treated at 80°C for 30 minutes and subsequently subjected to serial 10-fold dilutions. Aliquots of each dilution were spread onto agar plates and incubated at 37°C for 24 hours, after which single colonies were counted. Each replicate (three replicates per treatment group) was treated as an experimental unit for fecal bacterial analysis.

### Egg-laying performance

Daily egg-laying status was recorded for each hen, and the hen-housed egg production rate (HHEP) was calculated based on the cumulative number of eggs laid per replicate up to each time point using the following equation:

HHEP(%)=(Cumulativenumberofeggslaidperreplicateuptoeachtimepoint)/(Numberofhensatthestartoftestfeeding×numberofdays)×100

Daily egg weight was recorded for each replicate, and weekly mean egg weight and daily egg mass production were calculated. Feed intake was measured weekly for each replicate and converted to feed intake per hen per day. The feed conversion ratio (FCR) was calculated using the following equation:

FCR=Feedintakeperhenperday/Eggmassperhenperday

### Defective egg rate and eggshell strength measurement

The total number of defective eggs (broken eggs, shell-less eggs, misshapen eggs, double-yolk eggs, and small eggs) was recorded daily for each group. The defective egg rate was calculated as the percentage of defective eggs relative to the total number of eggs laid per group. Due to the low incidence of defective eggs, the analysis was conducted at the treatment group level.

Eggshell strength was measured using an eggshell strength tester (Fujihira Industry Co., Ltd.). Eggs were collected at the start of test diet feeding and every four weeks thereafter (five eggs per replicate at each sampling point). Measurements were performed after approximately one day of refrigerated storage.

### Fecal observation

The occurrence of soft feces and diarrhea was recorded for each hen twice daily (morning and evening). In the absence of a universally accepted classification of fecal consistency in laying hens, soft feces/diarrhea were defined as feces appearing slightly crumbled, crumbled or runny, following prior descriptions of fecal abnormalities in poultry (He*,* et al., 2022; Mozuriene*,* et al., 2024). The total number of events per bird at each sampling time was then summed for each replicate. Each replicate was treated as an experimental unit for the analysis of fecal abnormalities.

### Morphological analysis of the intestines

At eight time points (1, 2, 3, 5, 7, 9, 11, and 14 days after the end of test feed administration), one hen per replicate (three hens per treatment group) was euthanized by decapitation, and a 3-cm intestinal segment was collected from approximately 2–3 cm distal to the midpoint of the duodenum and ileum. The tissues were fixed in 10 % neutral-buffered formalin, processed routinely, and stained with hematoxylin and eosin (H&E). Villus height (μm) and crypt depth (μm) were measured under a light microscope, and the villus height-to-crypt depth ratio (VCR) was calculated.

### Statistical analysis

All statistical analyses were performed using EZR (version 1.68; Saitama Medical Center, Jichi Medical University, Japan) and R software (version 4.5.2; R Foundation for Statistical Computing, Vienna, Austria) (Kanda, 2013).

For MDA concentrations and weekly measurements of hen-housed egg production rate (HHEP), egg mass, feed intake, and feed conversion ratio (FCR), differences among treatment groups were evaluated at each time point using one-way ANOVA, followed by Tukey’s post hoc test. When assumptions of normality or homogeneity of variance were not met, data were analyzed using the Kruskal–Wallis test with Dunn’s post hoc test.

For intestinal morphological parameters (villus height-to-crypt depth ratio), differences among treatment groups at each sampling day were analyzed using one-way ANOVA, with individual hens treated as independent experimental units. In addition, the area under the curve from day 1 to day 14 (AUC1–14) was calculated for each hen to assess overall temporal responses. AUC values among treatment groups were compared using one-way ANOVA followed by Tukey’s post hoc test.

Statistical significance was set at *p* < 0.05, and 0.05 ≤ *p* < 0.10 was considered to indicate a trend.

### Experimental units and replication

The experimental unit was defined for each measured parameter according to the study design. For production performance parameters (e.g., egg production, egg mass, feed intake, and feed conversion ratio), each replicate (15 hens) was considered the experimental unit. For eggshell strength, individual eggs were measured, and the mean value per replicate at each sampling point was used for analysis. For intestinal morphology, measurements from multiple fields were averaged for each individual hen, and each hen was considered the experimental unit. For blood parameters, six hens per treatment group were repeatedly sampled over time, and individual hens were treated as experimental units in a repeated-measures design. For fecal bacterial counts, data were collected and analyzed at the replicate level, and each replicate was treated as the experimental unit. For fecal observations, measurements were recorded for each hen and then summarized at the replicate level, with each replicate treated as the experimental unit.

The number of samples used for each analysis was as follows: blood parameters were measured in two hens per replicate (six hens per treatment group), and the same individuals were repeatedly sampled at each time point. Intestinal morphology was analyzed in one hen per replicate (three hens per treatment group). Eggshell strength was measured using five eggs per replicate (15 eggs per treatment group) at each sampling point. Production performance parameters were calculated at the replicate level using three replicates per treatment group.

## Results and discussion

### Dietary supplementation with natto fermented by *Bacillus subtilis* var. *natto* strain TTCC903 mitigates heat stress

**Fig. 2:**
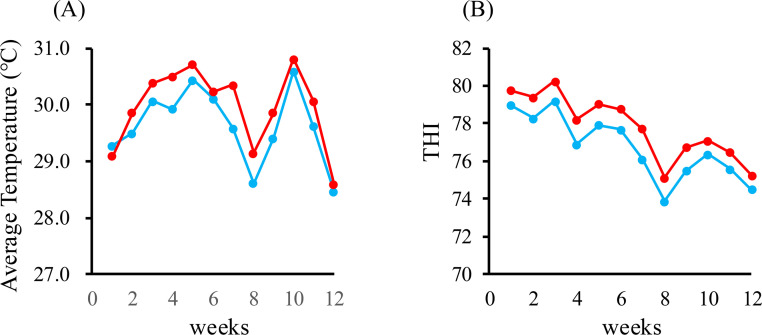
Environmental heat stress indices during the 12-week experimental period. (A) Weekly changes in temperature. (B) Weekly changes in the Temperature–Humidity Index (THI). Blue line: control group (non-supplemented group), red lines: natto-supplemented groups (0.05 % and 0.5 %).

Temperature changes during the experimental period were recorded weekly. Ambient temperatures fluctuated around 30°C and frequently exceeded 30°C during weeks 3–6, indicating sustained exposure to heat-stress conditions (Fig. 2A). Environmental heat stress was assessed using the temperature–humidity index (THI). The weekly average THI ranged from 74 to 80, corresponding to the Alert to Danger Zones throughout the 12-week period (Fig. 2B). Although air temperature showed minimal variation, a modest decline in humidity led to slightly lower THI values during weeks 9–12. Overall, these results indicate that the hens were consistently exposed to mild to moderate heat stress, which likely influenced feed intake and egg production.

**Fig. 3:**
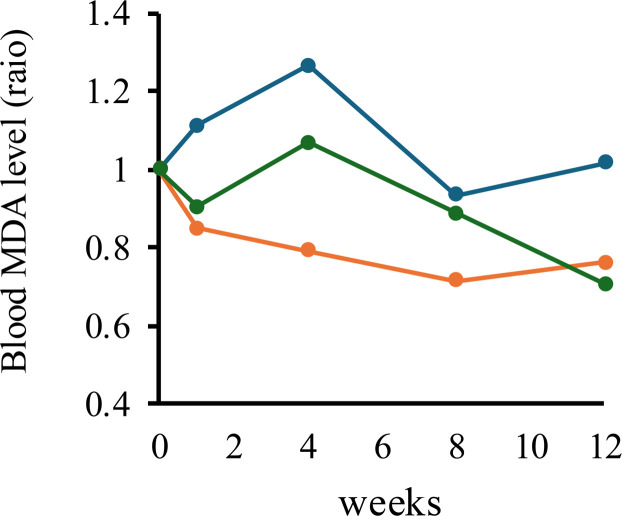
Temporal changes in plasma malondialdehyde (MDA) concentrations in aged laying hens under heat-stress conditions. MDA concentrations were measured in six hens per treatment group over time. Control group (blue), 0.05 % natto-supplemented group (orange), and 0.5 % natto-supplemented group (green).

Fig. 3 presents the blood malondialdehyde (MDA) levels in hens fed diets containing natto fermented with *B. subtilis* var. *natto* strain TTCC903 during the experimental period. MDA, the end product of lipid peroxidation, is widely used as a marker of oxidative stress (Ayala*,* et al., 2014). Exposure of poultry to high ambient temperatures is known to elevate MDA concentrations, and increases exceeding 20–50 % have been reported to indicate a strong heat stress response (Akbarian*,* et al., 2016; Altan*,* et al., 2003; Miao*,* et al., 2020). In the control group (without natto supplementation), MDA levels increased over time, peaking at 26 % above baseline. This finding indicates that, consistent with the environmental indices of heat stress, pronounced physiological oxidative stress was occurring. By contrast, in the natto-supplemented groups (0.05 % and 0.5 %), MDA concentrations showed differences, remaining stable or tending to decrease throughout the experimental period. Notably, in the 0.05 % natto-supplemented group, MDA concentrations decreased significantly, with a maximum reduction of 29 % (*p* < 0.05). Collectively, these results suggest that natto fermented with *B. subtilis* var. natto strain TTCC903 may contribute to the modulation of oxidative stress under heat stress conditions, as reflected by the observed changes in MDA levels. However, broader effects on heat stress responses were not directly evaluated in this study.

**Fig. 4:**
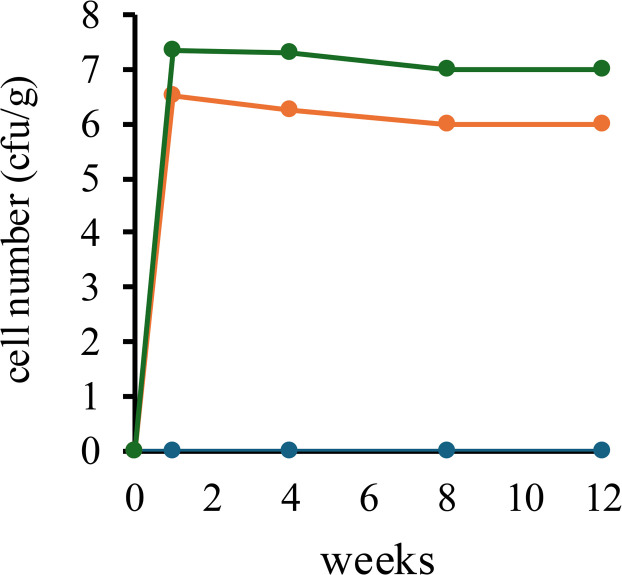
Changes in fecal counts of *B. subtilis* var. *natto* strain TTCC903 in aged laying hens during dietary supplementation under heat-stress conditions. Control group (blue), 0.05 % natto-supplemented group (orange), and 0.5 % natto-supplemented group (green).

The observed reduction in heat stress may be attributable to the probiotic effects of natto fermented with *B. subtilis* var. *natto* strain TTCC903. To verify intestinal colonization, bacterial counts were determined in fecal samples. In the 0.05 % and 0.5 % natto-supplemented groups, bacterial counts were 10^6^ and 10^7^ CFU/g, respectively, and remained stable during the supplementation period (Fig. 4). In contrast, no *B. subtilis* var. *natto* strain TTCC903 was detected in fecal samples from the control group. Moreover, the strain remained detectable in fecal samples during the 14-day observation period following the cessation of supplementation. Taken together, these results suggest that *B. subtilis* var. *natto* strain TTCC903 may alleviate heat stress through intestinal colonization and probiotic activity.

### Improvement in egg production with natto fermented by *B. subtilis* var. *natto* strain TTCC903 under heat stress

**Fig. 5:**
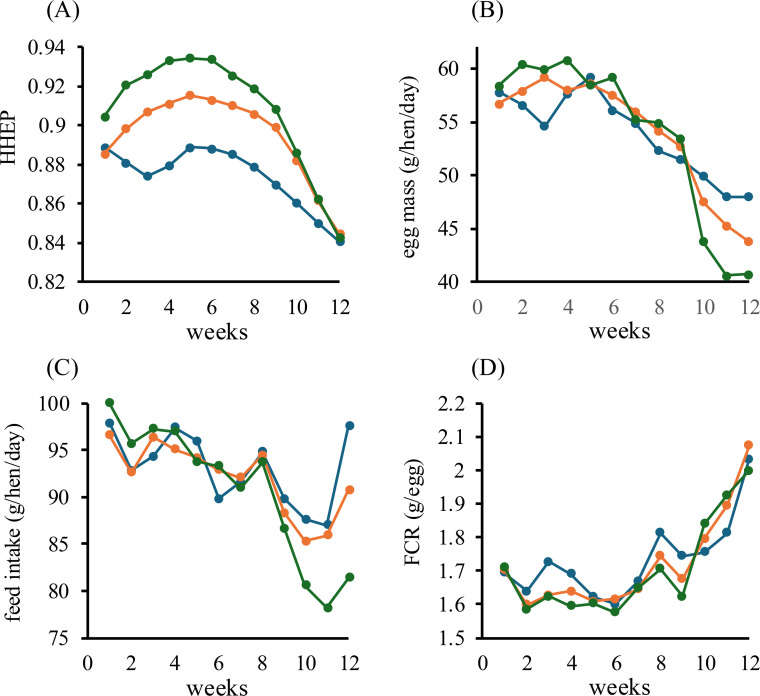
Effects of dietary supplementation with natto fermented by Bacillus subtilis var. natto strain TTCC903 on egg production performance in aged laying hens under heat-stress conditions. (A) Hen-housed egg production rate (HHEP, calculated based on cumulative egg production). (B) Egg mass (g/hen/day). (C) Feed intake (g/hen/day). (D) Feed conversion ratio (FCR). Blue: control group; orange: 0.05 % natto-supplemented group; green: 0.5 % natto-supplemented group.

In aged laying hens, the effects of natto supplementation on egg production performance were evaluated. All production performance parameters were analyzed at the replicate level (*n* = 3 replicates per treatment group). The hen-housed egg production rate (HHEP) was calculated and compared between the control and natto-supplemented groups (Fig. 5A). Between 20 and 60 weeks of age, the average HHEP typically ranges from 90 % to 95 %, and it is well established that egg production gradually declines thereafter with advancing age (Attia*,* et al., 2020; Bain*,* et al., 2016; Hy-Line_International, 2023). Heat stress further reduces HHEP compared with normal temperature conditions (Lara and Rostagno, 2013; Mignon-Grasteau*,* et al., 2015). In this experiment, under heat stress, the HHEP of hens without natto supplementation, calculated based on cumulative egg production, averaged 87.4 % across the study period. In contrast, HHEP in natto-supplemented hens averaged 89.5 % in the 0.05 % group and 90.8 % in the 0.5 % group. The 0.5 % group was significantly higher than the control (*p* < 0.05), whereas the 0.05 % group showed a tendency toward improvement (0.05 ≤ *p* < 0.10). Notably, during weeks 2–8, when THI values were relatively high, HHEP in the 0.5 % group exceeded 92.8 %. These results suggest that natto fermented with *B. subtilis* var. *natto* strain TTCC903 may enhance egg production performance.

Egg mass (g/hen/day) is summarized in Fig. 5B. Under typical rearing conditions, production in the late laying stage (≥70 weeks of age) generally ranges from 49 to 55 g/hen/day (Hy-Line_International, 2023), and decreases under high-temperature conditions (Arulnathan*,* et al., 2024; Mignon-Grasteau*,* et al., 2015). In this study, the control group averaged 53.9 g/hen/day, while the 0.05 % and 0.5 % groups averaged 54.0 and 53.9 g/hen/day, respectively, indicating little difference. However, during weeks 2–4 of the supplementation period, when THI values were highest, the non-supplemented group averaged 56.3 g/hen/day, compared with 58.7 and 60.4 g/hen/day in the 0.05 % and 0.5 % groups, respectively. Although not statistically significant, these results demonstrate a clear upward trend and suggest that natto supplementation may enhance egg production.

Feed intake (g/hen/day) is presented in Fig. 5C. Both the control and natto-supplemented groups exhibited a decreasing trend, with a maximum reduction of approximately 10 %. This uniform decrease is likely attributable to heat stress (Lara and Rostagno, 2013; Wasti*,* et al., 2020), indicating that natto supplementation does not influence feed preference. In broilers, probiotic supplementation under heat stress has been associated with 8 % lower feed intake relative to controls (Hernandez-Coronado*,* et al., 2025). In contrast, supplementation with natto fermented by *B. subtilis* var. *natto* strain TTCC903 did not further suppress feed intake, supporting its potential as an effective probiotic.

Feed conversion ratio (FCR) values are shown in Fig. 5D. At peak laying age (30–50 weeks), FCR values typically lie around 1.8–2.0, whereas in older hens (≥60 weeks) they tend to increase beyond 2.0, reflecting reduced production efficiency (Molnar*,* et al., 2017; Muir*,* et al., 2022). Under high-temperature rearing, reduced feed intake and heat-related physiological costs associated with heat stress can further exacerbate FCR (Molnar*,* et al., 2017; Muir*,* et al., 2022). In this study, the mean FCR values during the experimental period remained largely unchanged at 1.70–1.73 across all groups. However, during weeks 2–4 of the supplementation period, when THI values were highest, the control group showed no change from the baseline value of 1.69, whereas the 0.05 % group showed a non-significant but suggestive improvement to 1.62 (0.05 < *p* < 0.1). In the 0.5 % group, the FCR improved significantly to 1.60 (*p* < 0.05). These findings suggest that dietary supplementation with natto fermented by *B. subtilis* var. *natto* strain TTCC903 may improve feed efficiency.

Finally, supplementation with natto fermented by *B. subtilis* var. *natto* strain TTCC903 improved egg production efficiency in laying hens under heat stress. These results are consistent with previous reports of the beneficial effects of this strain in other animals, including enhanced growth and reduced cortisol levels in carp (Niwa*,* et al., 2026). Collectively, these findings indicate that supplementation with natto fermented by *B. subtilis* var. *natto* strain TTCC903 may alleviate heat stress and improve productivity in poultry under heat stress conditions.

### Reduction of defective eggs by natto fermented with *B. subtilis* var. *natto* strain TTCC903

Egg quality improvements following supplementation with natto fermented by B. subtilis var. natto strain TTCC903 were evaluated. Defective egg rate was calculated at the treatment group level by dividing the total number of defective eggs by the total number of eggs produced within each group. Because of the low incidence of defective eggs, the analysis was conducted at the treatment group level. Under normal conditions, defective eggs account for approximately 2–5 % in healthy flocks, whereas this rate can increase to 5–10 % under heat stress (Cheng and Ning, 2023; Kim*,* et al., 2024; Oluwagbenga and Fraley, 2023).

**Fig. 6:**
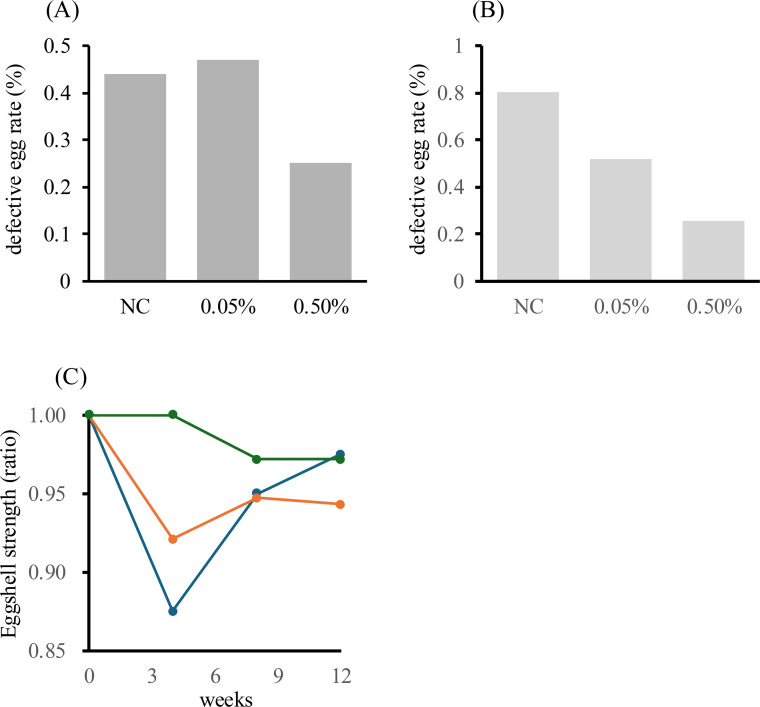
Effects of dietary supplementation with natto fermented by *B. subtilis* var. *natto* strain TTCC903 on defective egg rate and eggshell strength in aged laying hens under heat-stress conditions. (A) Percentage of defective eggs during the 12-week feeding period. (B) Defective egg rate during the early phase (weeks 1–4). (C) Temporal changes in eggshell strength. Blue: control group; orange: 0.05 % natto-supplemented group; green: 0.5 % natto-supplemented group.

The 0.5 % supplemented group showed an average defective egg rate of 0.25 % compared with 0.44 % in the control group, suggesting a tendency toward reduced defective egg incidence (Fig. 6A). However, because the baseline levels were already low, the biological significance should be interpreted with caution. Although no statistically significant differences were observed, the proportion of defective eggs showed a decreasing trend during the initial phase of natto supplementation (weeks 1-4), when differences in egg production capacity were noted (non-supplemented group: 0.81 %; 0.05 % group: 0.52 %; 0.5 % group: 0.25 %) (Fig. 6B). Although the absolute reduction in the defective egg rate was small, natto supplementation resulted in a relative improvement of approximately 50 %, which may be economically meaningful in large-scale commercial poultry production.

Next, eggshell strength was assessed. It is well established that eggshell strength decreases under heat stress, primarily due to reduced feed intake, impaired calcium absorption, and respiratory alkalosis (Brugaletta*,* et al., 2022; Cornescu*,* et al., 2023; Gonzalez*,* et al., 2025; Ribeiro*,* et al., 2025; Wasti*,* et al., 2020). In the control group, eggshell strength decreased by approximately 10 % after 4 weeks of feeding. In contrast, no reduction in eggshell strength was observed in the 0.5 % natto-supplemented group (Fig. 6C). These findings are consistent with previous reports showing that probiotic supplementation can improve eggshell strength and egg quality in laying hens (Onbasilar*,* et al., 2025). This effect may be associated with potential mechanisms of natto, including improvements in the intestinal environment and possible maintenance of calcium absorption efficiency. In addition, vitamin K₂ production and antioxidant activity may also contribute; however, these mechanisms were not directly investigated in the present study and should be considered speculative.

### Health-related effects of dietary natto fermented with *B. subtilis* var. *natto* strain TTCC903 in aged hens

Under high-temperature conditions, feed intake decreases and intestinal permeability increases, along with disruption of the intestinal microbiota. Fluid balance is disturbed and intestinal water absorption is impaired; simultaneously, elevated temperatures induce oxidative stress and inflammation in the intestinal mucosa. Together, these alterations increase the risk of gastrointestinal symptoms, including diarrhea (wet droppings), compared with thermoneutral conditions (Lara and Rostagno, 2013; Liu*,* et al., 2023; Oke*,* et al., 2024; Ruff*,* et al., 2020; Wasti*,* et al., 2020).

**Fig. 7:**
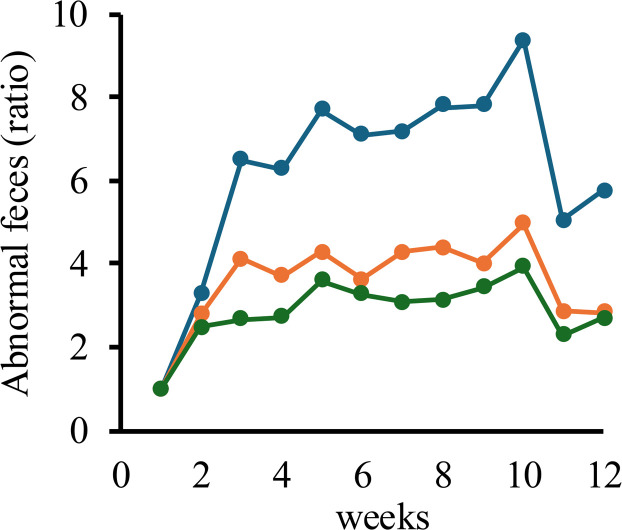
Incidence of diarrhea (wet droppings) in aged laying hens during the 12-week feeding trial under heat-stress conditions. Blue: control group; orange: 0.05 % natto-supplemented group; green: 0.5 % natto-supplemented group. Values represent the proportion of hens exhibiting soft feces or diarrhea at each time point.

Fecal observations were analyzed at the replicate level (*n* = 3 replicates per treatment group). In the present study, natto supplementation attenuated the increase in soft feces and diarrhea. In the control group, the incidence of soft feces and diarrhea began to rise in the first week of heat stress and was nine-fold higher by week 10 compared with week 0, consistent with previous reports. By contrast, in both the 0.05 % and 0.5 % natto-supplemented groups, the incidence was only four-fold higher compared with week 0, indicating mitigation of heat stress–related gastrointestinal symptoms (Fig. 7).

High ambient temperature compromises the intestinal barrier in chickens, leading to villus atrophy, crypt hyperplasia, and a reduced villus height–to–crypt depth ratio (VCR) (Kikusato and Toyomizu, 2023; Patra and Kar, 2021; Rostagno, 2020). Under thermoneutral conditions, representative VCR values are approximately 7–8 in the duodenum and 5 in the ileum, but these values typically decrease under heat stress, indicating impaired absorptive capacity and increased intestinal permeability (Lei*,* et al., 2015). However, evidence in older laying hens (≥70 weeks) remains limited, despite their greater susceptibility to heat stress. We therefore hypothesized that dietary supplementation with *B. subtilis* var. *natto* strain TTCC903 fermented natto would attenuate heat stress-induced intestinal damage and reduce the risk of diarrhea (wet droppings) by preserving VCR. Accordingly, this study evaluated segment-specific changes in VCR (primary endpoint: ileum; secondary: duodenum) and gastrointestinal symptoms in aged hens under field-relevant heat stress.

**Fig. 8:**
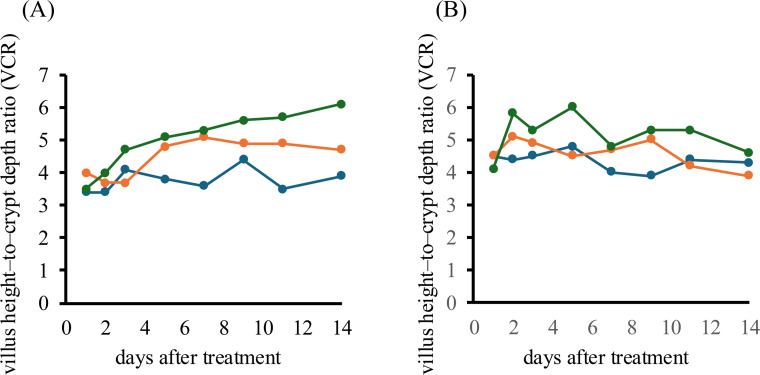
Effects of dietary supplementation with natto fermented by *B. subtilis* var. *natto* strain TTCC903 on intestinal morphology in aged laying hens under heat-stress conditions. (A) Villus height-to-crypt depth ratio (VCR) in the ileum. (B) Villus height-to-crypt depth ratio (VCR) in the duodenum. Blue: control group; orange: 0.05 % natto-supplemented group; green: 0.5 % natto-supplemented group.

The ileal VCR (mean per hen) increased in a dose-dependent manner with natto supplementation (Fig. 8A). The period mean (days 1–14) was 3.76 (control), 4.48 (0.05 %), and 5.00 (0.5 %); day-14 values were 3.9, 4.7, and 6.1, respectively. The baseline-adjusted change (Δ14-1) was +0.5, +0.7, and +2.6; the linear slope (b/day) was +0.025, +0.090, and +0.180; and AUC_1–14_​ -the area under the time – VCR curve from day 1 to 14 (units: VCR·day) - was 49.45, 60.15, and 68.20, respectively. For AUC_1–14_​, the 0.05 % group exhibited numerically higher values than the control group, indicating a non-significant trend toward improvement, whereas the 0.5 % group showed a sustained and statistically significant improvement (*p* < 0.05). The period-mean analysis showed the same ranking (0.5 % > 0.05 % > control), corroborating the AUC-based findings. Thus, dietary supplementation with natto fermented by *B. subtilis* var. *natto* strain TTCC903 improved ileal condition under heat stress.

In the duodenum, the 0.5 % group showed the highest period mean (5.15) and AUC_1–14_ (68.15) (Fig. 8B). Although minor fluctuations occurred within the period, VCR values remained predominantly above baseline across the observation window, and the combination of the largest AUC with a non-negative overall slope supports a sustained effect. By contrast, the 0.05 % group exhibited an early rise followed by a decline, peaking in the first few days and then falling to day-14 = 3.9 with Δ(14–1) = −0.6 and a negative slope (−0.058/day), indicating a transient response. The control group remained relatively stable (day-14 4.3, AUC_1-14_ 56.25). Consistent with these results, duodenal outcomes likewise showed a trend toward amelioration of heat stress-related changes with natto supplementation, mirroring the pattern seen in the ileum.

These findings suggest that natto supplementation was associated with an improved VCR, particularly in the ileum, indicating a morphological improvement of the intestinal mucosa. The effect is consistent with (but does not by itself prove) microbiota modulation under heat stress (Idowu*,* et al., 2025; Luo*,* et al., 2023; Ncho, 2025), and improved mucosal architecture could plausibly enhance water absorption; however, this was not directly evaluated in the present study. Similar improvements in intestinal morphology, including increases in villus height and villus height-to-crypt depth ratio, have been reported following dietary supplementation with plant-derived bioactive compounds and organic acids in poultry (Onbaşılar*,* et al., 2025), supporting the interpretation that the observed changes reflect a general morphological response of the intestinal mucosa. Moreover, a healthier intestinal environment may support calcium uptake, potentially contributing—together with other factors—to improved eggshell strength observed elsewhere in this study; however, this interpretation remains speculative and the underlying mechanisms were not directly investigated.

## Conclusion

In this study, supplementation with natto fermented by *B. subtilis* var. *natto* strain TTCC903 mitigated heat stress and improved egg production performance in aged laying hens. These effects were not uniform across the entire trial but were most pronounced during periods of elevated environmental stress, suggesting that the probiotic efficacy of this natto product may be influenced by environmental conditions. Moreover, while beneficial effects of *B. subtilis* var. *natto* strain TTCC903 have previously been reported in fish, the present study is significant as the first to demonstrate its potential applicability in poultry. Previous reports of antiviral activity of this strain further suggest that it may have additional biological functions, although these effects were not examined in the present study (Kobayashi*,* et al., 2023; Oba*,* et al., 2021).

Previous studies have reported improved productivity and fermentation in ruminant livestock following the addition of *B. natto* (Chang*,* et al., 2021; Sun*,* et al., 2013). Furthermore, studies in poultry have demonstrated that the efficacy of probiotics tends to be amplified under environmental conditions such as heat stress (Hashemitabar and Hosseinian, 2024; Sayed*,* et al., 2025). Future research should clarify whether these effects are specific to *B. subtilis* var. *natto* strain TTCC903 or represent a common property of natto bacteria in general. This will advance the development of probiotic technology for widespread adoption in the livestock industry.

## Ethical approval

This experiment was conducted in accordance with the Animal Experiment Guidelines of the Japan Scientific Feeds Association, approved by the Animal Experiment Control Committee of the Japan Scientific Feeds Association (Approval No. R06-08), and performed appropriately.

## CRediT authorship contribution statement

**Kaori Suzuki:** Writing – review & editing, Writing – original draft, Formal analysis, Data curation, Conceptualization. **Hikaru Ikarugi:** Writing – review & editing, Methodology, Formal analysis, Data curation. **Takanobu Nishikawa:** Writing – review & editing, Supervision, Funding acquisition, Formal analysis, Data curation, Conceptualization. **Ryosuke Kadoya:** Writing – review & editing, Writing – original draft, Supervision, Methodology, Formal analysis, Data curation, Conceptualization.

## Disclosures

This study was conducted in collaboration with Takanofoods Co., Ltd. All experiments, data analyses, and interpretations were conducted independently by the authors. The authors declare that there are no conflicts of interest that could have influenced the outcomes of this study.
